# Host Ecology Rather Than Host Phylogeny Drives Amphibian Skin Microbial Community Structure in the Biodiversity Hotspot of Madagascar

**DOI:** 10.3389/fmicb.2017.01530

**Published:** 2017-08-17

**Authors:** Molly C. Bletz, Holly Archer, Reid N. Harris, Valerie J. McKenzie, Falitiana C. E. Rabemananjara, Andolalao Rakotoarison, Miguel Vences

**Affiliations:** ^1^Zoological Institute, Technical University of Braunschweig Braunschweig, Germany; ^2^Department of Biology, James Madison University, Harrisonburg VA, United States; ^3^Department of Ecology and Evolutionary Biology, University of Colorado Boulder Boulder, CO, United States; ^4^Mention Biologie et Biodiversité Animale, University of Antananarivo Antananarivo, Madagascar

**Keywords:** host-associated microbiota, 16S rRNA illumina sequencing, amphibians, community assembly, bacteria

## Abstract

Host-associated microbiotas of vertebrates are diverse and complex communities that contribute to host health. In particular, for amphibians, cutaneous microbial communities likely play a significant role in pathogen defense; however, our ecological understanding of these communities is still in its infancy. Here, we take advantage of the fully endemic and locally species-rich amphibian fauna of Madagascar to investigate the factors structuring amphibian skin microbiota on a large scale. Using amplicon-based sequencing, we evaluate how multiple host species traits and site factors affect host bacterial diversity and community structure. Madagascar is home to over 400 native frog species, all of which are endemic to the island; more than 100 different species are known to occur in sympatry within multiple rainforest sites. We intensively sampled frog skin bacterial communities, from over 800 amphibians from 89 species across 30 sites in Madagascar during three field visits, and found that skin bacterial communities differed strongly from those of the surrounding environment. Richness of bacterial operational taxonomic units (OTUs) and phylogenetic diversity differed among host ecomorphs, with arboreal frogs exhibiting lower richness and diversity than terrestrial and aquatic frogs. Host ecomorphology was the strongest factor influencing microbial community structure, with host phylogeny and site parameters (latitude and elevation) explaining less but significant portions of the observed variation. Correlation analysis and topological congruency analyses revealed little to no phylosymbiosis for amphibian skin microbiota. Despite the observed geographic variation and low phylosymbiosis, we found particular OTUs that were differentially abundant between particular ecomorphs. For example, the genus *Pigmentiphaga* (Alcaligenaceae) was significantly enriched on arboreal frogs, *Methylotenera* (Methylophilaceae) was enriched on aquatic frogs, and *Agrobacterium* (Rhizobiaceae) was enriched on terrestrial frogs. The presence of shared bacterial OTUs across geographic regions for selected host genera suggests the presence of core microbial communities which in Madagascar, might be driven more strongly by a species’ preference for specific microhabitats than by the physical, physiological or biochemical properties of their skin. These results corroborate that both host and environmental factors are driving community assembly of amphibian cutaneous microbial communities, and provide an improved foundation for elucidating their role in disease resistance.

## Introduction

Mucosal environments of vertebrate hosts are inhabited by diverse microbial assemblages (Bäckhed et al., 2005; Rosenthal et al., 2011; Krediet et al., 2013; Brune and Dietrich, 2015; Colombo et al., 2015; Jiménez and Sommer, 2016). These communities often play critical roles in host development and in maintaining host health (Stecher and Hardt, 2008; Robinson et al., 2010; Engel and Moran, 2013; Sanford and Gallo, 2013; Fraune et al., 2014). With the advent of next generation sequencing, it is possible to study host microbiota in intricate detail, and numerous host microbiotas have been characterized (Caporaso et al., 2011). Most studies to date, however, concentrate on human and other mammalian systems, and our ecological understanding of host microbiota from a diverse host range is still in its infancy (Robinson et al., 2010; Fierer et al., 2012).

Amphibian skin hosts one of the best-studied wildlife microbiotas due to the role of these cutaneous microbial communities in meditating defense against the lethal pathogen, *Batrachochytrium dendrobatidis* (*Bd*) (Belden and Harris, 2007; Bletz et al., 2013; Jiménez and Sommer, 2016). These microbial communities provide a first line of defense against invading pathogens, such as *Bd* (Becker and Harris, 2010). This fungal pathogen causes the disease chytridiomycosis, which is responsible for amphibian declines around the world, particularly in Central America, Australia, and the western US (Berger et al., 1998; Lips et al., 2006; Cheng et al., 2011). Bacterial symbionts isolated from amphibian skin can inhibit *Bd* growth through the production of anti-fungal compounds (Harris et al., 2006; Brucker et al., 2008a,b; Flechas et al., 2012; Woodhams et al., 2015), and population survival has been linked to the proportion of amphibians with *Bd-*inhibitory bacteria in the western United States (Lam et al., 2010). Microbial therapies have been proposed as a possible disease mitigation strategy for combating chytridiomycosis (Bletz et al., 2013; Walke and Belden, 2016; Woodhams et al., 2016), and thus, investigation of the basic ecological principles dictating skin microbial community structure on amphibians can inform the development and application of probiotic therapies.

The extent to which host factors versus environmental factors structure skin microbial communities of amphibians as well as those of other hosts is not fully understood. Furthermore, no studies to date have explored the role of host phylogeny (i.e., do amphibian skin microbiotas exhibit phylosymbiosis)? (Brooks et al., 2016) or the role of host ecology in shaping amphibian skin microbial communities on a large scale. While multiple studies have demonstrated that amphibian cutaneous microbiotas vary among species (McKenzie et al., 2012; Kueneman et al., 2014; Belden et al., 2015), most studies are limited to a few host species and often focus on hosts with distinct host ecologies (e.g., arboreal versus terrestrial). Physical and chemical properties of the skin ecosystem likely differ between amphibian species. For example, amphibian species produce different suites of antimicrobial peptides (Woodhams et al., 2006; Conlon, 2011), and alkaloids are synthesized or sequestered by particular amphibian species (Erspamer, 1994; Daly, 1995). Species may also differ in the mucins and glycoproteins present on their skin (Austin, 2000; Wells, 2007). Factors such as these, all may play a role in shaping the cutaneous microbiota of amphibians. On the other hand, species also differ in their ecology, and thus spend time in different micro-habitats, exposing them to different microbial reservoirs or pathogenic stressors. This variation in the microbes available for colonization and/or the pathogenic stressors could also be a strong force dictating community composition.

Madagascar is an amphibian biodiversity hotspot, home to over 400 endemic frog species (Vences et al., 2009; Vieites et al., 2009). Multiple locations are known to have over 100 co-occurring species, which is ideal for investigating the primary drivers of microbial community assembly on amphibian skin on a large scale and teasing apart how symbiotic microbiota are influenced by environmental factors, host-produced factors, and host ecology. Therefore, using this system, we explored the factors structuring the cutaneous microbial communities of amphibians in Madagascar, by investigating the following main questions: (1) what are the primary drivers of microbial community structure and diversity?, and more specifically, (2) what is the role of host phylogeny *versus* host ecology in shaping microbial community structure and diversity?

## Materials and Methods

### Field Sampling

**FIGURE 1:**
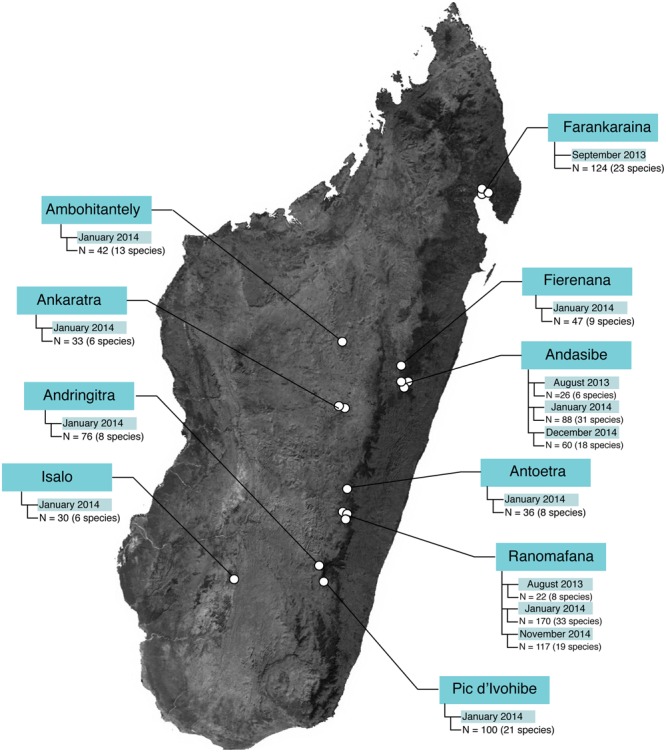
Sample sizes across study locations in Madagascar. The base map was obtained from www.worldofmaps.net. No permission is required from the copyright holders for the reproduction of this image. Points on the map were generated using Google Earth Pro and afterwards edited in Adobe^®^ Illustrator^®^ CS6 software.

Field sampling occurred during three field visits: 14 August – 12 September 2013, 4 January – 9 February 2014, and 5 November – 15 December 2014. In total, 1021 microbial samples (989 frog skin swabs, 32 environmental samples) were collected from 10 locations (30 sites) and 96 host species (**Figure 1** and Supplementary Tables 1, 2).

Amphibians were captured during day and night surveys with clean nitrile gloves and placed in sterile Whirl-Pak^®^ bags (Nasco, Fort Atkinson, WI, United States). For skin microbe sampling, individuals were removed from the bag with a clean pair of nitrile gloves and rinsed with 50 ml of sterilized water. After rinsing, each individual was swabbed with a single sterile rayon swab (MW113, Medical Wire Equipment & Co. Ltd., Corsham, United Kingdom), applying 10 strokes on the ventral abdomen, 5 strokes on each ventral thigh, and 5 strokes on each foot. Swabs were stored in microcentrifuge tubes and transported on ice until transfer to a -20°C freezer. Environmental samples were collected from amphibian-associated habitat, including soil, water, and leaves. For soil samples, 1–2 g of soil were collected; for water samples, 60–120 ml of water were hand-pumped through a 0.22 μm filter; for leaf samples, the surface was swabbed with 30 strokes. Environmental samples were stored in 2 ml tubes and transported on ice until transfer to a -20°C freezer. This study was approved by the Institutional Animal Care and Use Committee of James Madison University (protocol #A01-15), and necessary research and access permits were obtained from the Malagasy Direction Générale des Forêts (DGF) and Madagascar National Parks for all sampling.

### DNA Extraction and PCR

Bacterial DNA was extracted using the MoBio PowerSoil DNA isolation kit (MoBio Laboratories, Carlsbad, CA, United States) following the manufacturer’s protocol with minor modifications to increase DNA yield. The V4 region of the 16S rRNA gene was PCR-amplified in triplicate with barcoded primers (515f/806r) following Kueneman et al. (2014). Amplicon concentration was quantified with Quant-iT PicoGreen dsDNA Assay kit (Thermofisher, Waltham, MA, United States). Equal concentrations of each sample were pooled, and the pooled amplicons were cleaned using MoBio UltraClean PCR Clean-up kit (MoBio Laboratories, Carlsbad, CA, United States). The pooled barcoded amplicons were sequenced using 2 × 150 pair-end technology on an Illumina MiSeq platform at the BioFrontiers Institute at the University of Colorado.

### Sequence Processing

Sequence reads were filtered and pre-processed in Quantitative Insights Into Microbial Ecology (QIIME) (Caporaso et al., 2010). Forward reads were demultiplexed and filtered with the following criteria to retain only high quality reads: no Ns within the sequence, no barcode errors, and a minimum of three consecutive low-quality base pairs (minimum *q* = 10) before read truncation. Only forward reads were used because reverse reads typically suffer from lower quality (Kwon et al., 2013). Quality filtered sequences were clustered into operational taxonomic units (OTUs) using the deblur workflow^1^. Deblur is a new sub-operational taxonomic unit (sOTU) approach for amplicon sequencing that incorporates known Illumina error profiles and uses Hamming distances along with a greedy algorithm (Amir et al., 2017). Within this workflow, sequences were trimmed to 150 bp, and sOTU clusters (hereafter called OTUs) with less than 25 reads were removed. Taxonomy was assigned with Ribosomal Database Project Classifier (Wang et al., 2007), and a phylogenetic tree was built in QIIME using the fasttree algorithm (Price et al., 2010). Samples were subsequently rarefied at 4,000 reads per sample to normalize read counts across samples. Sequences have been archived in the SRA database (Bioproject accession number: PRJNA394790).

### Data Analysis

Number of OTUs, Chao1, effective number of species [exp(Shannon index), Jost, 2006], and Faith’s phylogenetic diversity were calculated for all samples as measures of alpha diversity, i.e., species richness and species diversity. General linear models (GLM) were used to test which factors were significant predictors of amphibian skin bacterial richness and diversity in SPSS v24 (IBM Corp, Armonk, NY, United States). The same factors described below for PERMANOVAs were included in the models to represent site parameters, host ecomorphology and host phylogeny.

Beta diversity was calculated as weighted and unweighted Unifrac distances in QIIME. The resulting matrices were used to explore patterns in beta diversity in two main ways: (1) Multiple Regression on distance Matrices (MRM), and (2) Permutational multivariate analysis of variance (PERMANOVA).

MRM was used to simultaneously explore the role of host phylogeny and host ecology on microbial community structure. We made distance matrices to represent (a) evolutionary divergences among host species (i.e., host phylogeny) and (b) ecomorphological differences (i.e., host ecology). Evolutionary divergences among host species (with 2 or more sampled individuals) were calculated as patristic phylogenetic distances from an ultrametric timetree. We first reconstructed a phylogenetic tree of all host species included in our study based on partial sequences of the mitochondrial 16S rRNA gene (Vieites et al., 2009), in MEGA7 (Kumar et al., 2016) under the Maximum Likelihood optimality criterion, with a general time-reversible (GTR + I) substitution model and using SPR branch swapping. We then manually corrected the tree topology for some wrongly reconstructed deep relationships, based on published multigene phylogenies of mantellids (Wollenberg et al., 2011), microhylids (Scherz et al., 2016), and intrafamilial relationships (Roelants et al., 2007). We then entered this topology as the usertree in MEGA7 along with the 16S alignment, and reconstructed an ultrametric tree using the Real-time method, without absolute calibration. Patristic pairwise distances among included amphibian species were calculated from this ultrametric tree in R using the ape and adephylo packages (Paradis et al., 2004; Jombart and Dray, 2008). Ecomorphological distances were calculated on the basis of major species traits that can be hypothesized to influence the cutaneous microbiota: (1) degree of arboreality, (2) degree of water dependence, (3) kind of breeding water body, (4) primary forest dependence, (5) reproductive mode, (6) aquatic or terrestrial egg deposition, and (7) body size (Glaw and Vences, 2007). The traits were coded as either ordered/ordinal (1,2,4,7) or unordered/categorical (3,5,6) characters. For a complete list of all character states see Supplementary Tables 3, 4. Distance matrices among species were calculated using PAUP v. 4b10 (Swofford, 2002). We considered *a priori* the first three traits as most likely to be important as they directly indicate distinct microhabitats that the frogs are exposed to, while we considered the remaining traits as possibly important, but probably less influential. MRM analyses were completed with the ‘ecodist’ package in R (Goslee and Urban, 2007; R Core Team, 2014), testing the correlation of the phylogenetic and ecomorphological matrices with microbial community structure for the full dataset (89 species) as well as for amphibians occurring at a single hyperdiverse site (38 species), Ranomafana. Microbial communities were represented by weighted Unifrac distance matrices derived from OTU tables averaged by frog species; that is, the rarified OTU table was first averaged by host species, and pair-wise weighted unifrac distances were subsequently calculated.

Topological congruency analysis was used to further explore the possible existence of phylosymbiosis. For this, we quantified congruence between the host phylogenetic tree and microbial dendrograms using the TreeCmp program. Microbial dendrograms were created in QIIME using both Weighted Unifrac and Bray-Curtis distances derived from OTU tables averaged by frog species. Using TreeCmp, we calculated the normalized Robinson–Foulds scores, where values of 0 indicates complete congruence and values of 1 indicate lack of congruence.

Because no topological congruence was found and the MRM analyses suggested a stronger correlation with host ecology than with phylogeny (see Results), we performed PERMANOVAs to further understand how frog ecomorphology as well as the other variables influence the skin bacterial communities. PERMANOVAs were completed in R (R Core Team, 2014) with the ADONIS2 function in the ‘vegan’ package (Oksanen et al., 2017) to test which factors significantly explained the observed variation in microbial community composition and structure. The “margin” option in ADONIS2 was used to assess marginal effects of each term in a model including all other variables. The following factors were included in the models: elevation, latitude, ecomorphological category [hereafter called host ecomorph, with three categories: (a) arboreal, (b) aquatic and semi-aquatic, or (c) terrestrial], as well as an approximate representation of host phylogeny. The phylogeny variable was chosen because the large number of frog species sampled made it infeasible to include host species as a categorical variable in any model, and because a categorical host species variable would not have captured the phylogenetic relationships among the amphibians. We, therefore, performed non-metric multidimensional scaling (nMDS) constrained to 1 dimension on the patristic distance matrix using SPSS v24 (IBM Corp, Armonk, NY, United States). The coordinates of the nMDS axis were subsequently extracted and used as a proxy variable in PERMANOVA models. PERMANOVA was also used to test whether frog skin microbiota differed from that of the environment in PRIMER7 (Clarke and Gorley, 2015).

Unweighted pair group method with arithmetic mean (UPGMA) was used to evaluate clustering patterns across host ecomorphs and Similarity Profile Analysis (SIMPROF) was used to statistically test for significant structure within the created UPGMA dendrogram. Both analyses were performed in PRIMER7 (Clarke and Gorley, 2015).

The Linear discriminant analysis Effect size (LEfSe) method (Segata et al., 2011) was used to identify which bacterial taxa were most likely explaining the observed differences between categories of interest. LEfSe was used to identify differentially abundant taxa between frogs and the environment, and also to identify differentially abundant taxa among host ecomorphs. Default parameters were used with the exception of increasing the LDA score; taxa with LDA scores greater than 3.0 were considered significant.

Bipartite networks were used to visualize the association of bacterial taxa with a given host ecomorph. These networks were calculated in R (Sedlar et al., 2016), and visualized with Gephi (Bastian et al., 2009).

## Results

### Malagasy Frog Skin Microbiota Differs from Environmental Substrates

Frog cutaneous microbial communities were less species rich than those of the environment (# of OTUs – Frog: 219.9 3 ± 6.71(SE)/Env: 948.96 ± 72.98; Chao1 – Frog: 265.37 ± 8.57/Env: 1415.79 ± 130.76), and also less diverse than that of the environment (Effective # of species – Frog: 509.8 ± 40.0/Env: 8644.5 ± 1337.8; Faith’s PD – Frog: 37.28 ± 0.81/Env: 106.86 ± 5.88).

**FIGURE 2:**
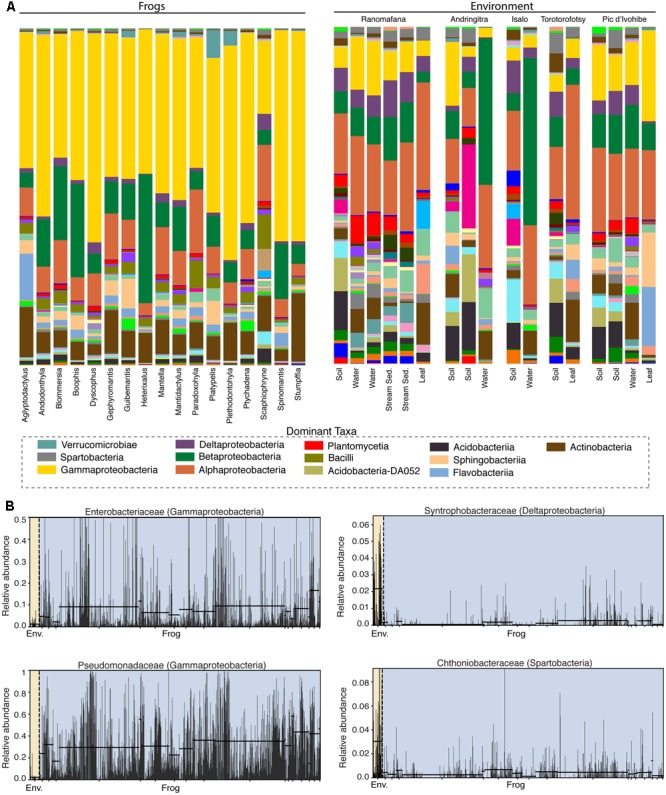
Bacterial composition differs between the skin of Malagasy frog hosts and the environment. **(A)** Taxonomic bar plots for bacterial communities on frogs (by host genus) and in the environment (by substrate type) at the class level. Dominant taxa are identified in the legend. **(B)** Four representative differentially abundant taxa identified by LEfSe analysis. Two taxa that exhibited greater relative abundance on frogs (left) and two taxa that exhibited greater relative abundance in the environment (right) are presented. Yellow highlighting indicates the environment and blue highlighting indicates frogs. Each bar represents an individual sample. Supplementary Table 2 presents all LEfSe-identified taxa with LDA Scores.

Frog skin microbiotas were dominated by Proteobacteria (*Gamma* – 46.6%, *Beta* – 15.4%, *Alpha* – 9.4%, *Delta* – 1.6%), Bacteriodetes (8.1%), Actinobacteria (7.9%), Firmicutes (3.6%), and Acidobacteria (2.3%). Microbial community structure on frog skin strongly differed from that of the environment (PERMANOVA: Pseudo-F = 33.34, *p* = 0.001, **Figure 2A**). Soil environments were comprised predominantly of Acidobacteria (19.7%), Proteobacteria (*Alpha* – 17.3%, *Gamma* – 8.7%, *Beta* – 8.5%, *Delta* – 6.4%), Bacteriodetes (7.3%), Verrucomicrobia (5.3%), Chloroflexi (5.1%) Actinobacteria (4.8%), and Planctomycetes (3.3%). Water environments were comprised of Proteobacteria (*Beta* – 23.7%, *Alpha* – 21.4%, *Gamma* – 9.5%, *Delta* – 3.9%), Actinobacteria (11.8%), Bacteriodetes (7.7%), Planctomycetes (3.9%), Firmicutes (3.0%), Acidobacteria (2.7%), and Verrucomicrobia (2.5%). Leaf surfaces were comprised of Proteobacteria (*Alpha* – 30.2%, *Gamma* – 12.3%, *Beta* – 5.2%, *Delta* – 2.8 %), Bacteriodetes (24.7%), Actinobacteria (10.1%), Acidobacteria (3.7%), Cyanobacteria (2.8%), and Verrucomicrobia (2.7%).

Fifty-eight bacterial taxa were identified as differentially abundant between frog hosts and the environment using the LEfSe method (LDA > 3). More specifically, 47 taxa exhibited greater relative abundance in the environment and 11 taxa exhibited greater relative abundance on frogs (Supplementary Table 5). For example, Synthrophobacteraceae (Deltaproteobacteria) and Chthoniobacteraceae (Spartobacteria) were enriched in the environment, while Pseudomonadaceae (Gammaproteobacteria) and Enterobacteriaceae (Gammaproteobacteria) were enriched on frog skin (**Figure 2B**).

### Drivers of Skin Microbial Composition, Structure and Diversity

To simultaneously explore the role of host phylogeny and host ecology we performed MRMs. When considering the entire dataset, both host phylogeny and host ecology were not significant (MRM -Phylo: *p* = 0.400, Eco: *p* = 0.515). However, when considering the single hyperdiverse site Ranomafana, host ecomorphology, derived from the 3-character matrix, was significant while host phylogeny was not (MRM – Eco: *p* = 0.029, Phylo: *p* = 0.579). Inclusion of additional ecological traits in the calculation of the ecomorphological distance matrix did not improve the correlation with weighted unifrac distances of the microbial communities (Supplementary Table 6), suggesting that arboreality, water dependence and breeding water body are the most important ecological traits influencing the cutaneous bacterial communities of Malagasy amphibians.

Topological congruency analysis also showed a lack of congruence between host phylogenetic trees and microbial dendrograms, suggesting a limited effect of host phylogeny (Full dataset – Weighted Unifrac: normalized RF score = 1, Bray-Curtis: normalized RF score = 0.98; Ranomafana-Weighted Unifrac: normalized RF score = 0.98, Bray-Curtis: normalized RF score = 1).

**FIGURE 3:**
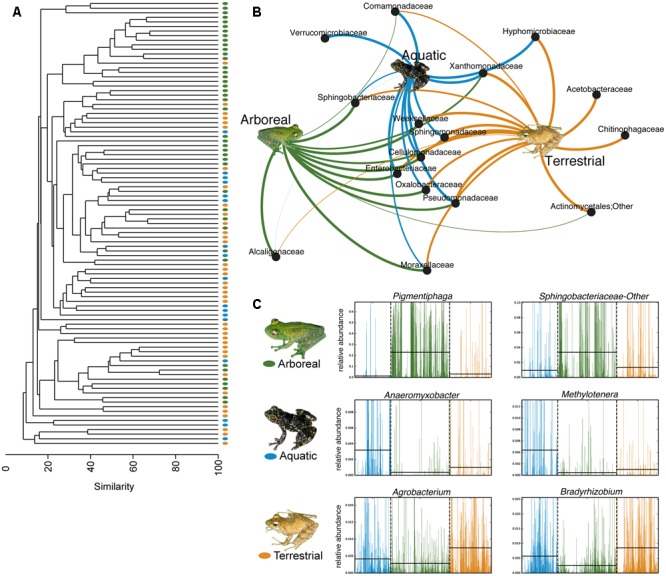
Host ecology affects amphibian skin microbial community structure and composition. **(A)** UPGMA clustering of the microbial communities on the 88 sampled frog species. Each point represents a frog species. **(B)** Bipartite network showing the association of particular bacterial taxa with certain host ecomorphs (analysis based on a 0.01 relative abundance threshold for OTU inclusion). Lines connect family level OTUs to host ecomorph categories and are weighted by relative abundance. **(C)** Six representative differentially abundant taxa identified by LEfSe analysis. Two taxa that exhibited greater relative abundance on frogs from each host ecomorph are presented. Green represents arboreal frog species, blue represents aquatic frog species, and yellow represents terrestrial frog species. Supplementary Table 4 presents all LEfSe-identified bacterial families. Inset frog photos were taken by M. Vences.

Using PERMANOVAs, host ecomorph was the strongest predictor of skin bacterial community structure [PERMANOVA – Weighted: Pseudo-*F*_(2,980)_ = 30.308, *p* = 0.001; Unweighted: Pseudo-*F*_(2,980)_ = 7.142, *p* = 0.001, **Table 1**]; however, host phylogeny (nMDS 1), latitude, and elevation also explained significant portions of the variation (**Table 1**). Pairwise comparisons of individuals within ecomorphs were lower than the pairwise distances between ecomorphs (Supplementary Table 7), and pairwise comparisons of host ecomorph classes showed that each ecomorph was significantly different from the others (Arb-Ter: *t* = 6.699, *p* = 0.001; Arb-Aqu *t* = 5.625, *p* = 0.001; Ter-Aqu *t* = 2.875, *p* = 0.001; **Figure 3**). Multivariate dispersion also did not differ between host ecomorphs (PERMDISP- *F* = 1.699, *p* = 0.223). In addition, UPGMA clustering showed that host species within the same ecomorph class typically clustered together (**Figure 3**), and SIMPROF analysis revealed that there was significant structure within the dendrogram (SIMPROF – *p* = 0.001).

**Table 1:** ADONIS results for main factors influencing beta diversity of cutaneous bacterial communities on Malagasy amphibians.

		Beta diversity metric	
		Weighted	Unweighted
Factor	DF	Unifrac	Unifrac
Host ecomorph	2 980	**30.308** **0.001**	7.142 0.001
Latitude	1 980	5.930 0.001	**8.359** **0.001**
Elevation	1 980	4.282 0.001	3.052 0.001
Host phylogeny (nMDS1)	1 980	5.034 0.001	4.057 0.001

*Model results for weighted and unweighted Unifrac matrices are provided. Pseudo-F *p*-values are presented. The strongest predictor is bolded.*

**FIGURE 4:**
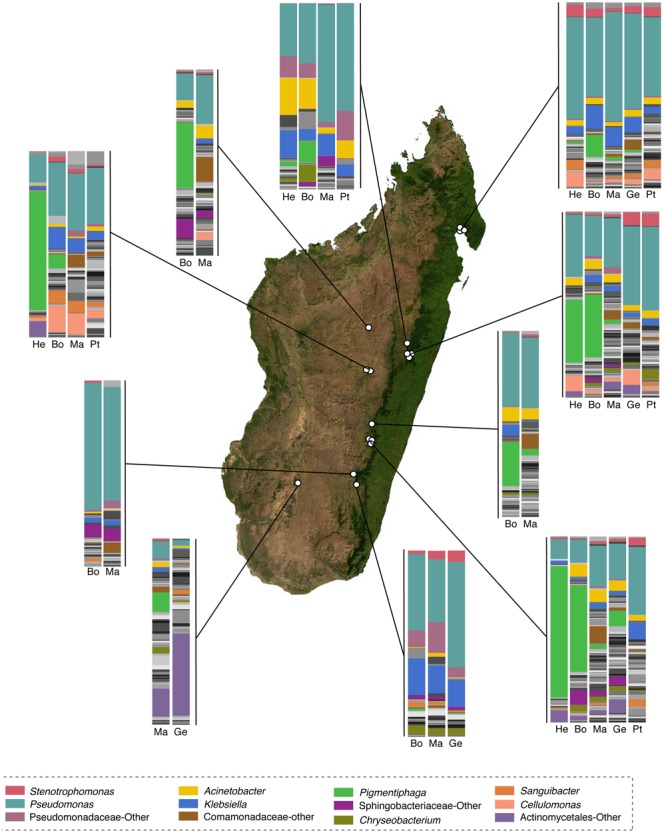
Taxonomic composition of skin microbiota of selected genera of Malagasy frogs across locations. Within the bar plots, major taxa are colored, with each color representing a bacterial genus. Other taxa are presented in gray scale. Bar plots represent all OTUs with relative abundances greater than 0.1% across the dataset. Host genera are abbreviated as follows: He, *Heterixalus*; Bo, *Boophis*; Ma, *Mantidactylus*; Ge, *Gephyromantis*; and Pt, *Ptychadena.* The base map was obtained from www.worldofmaps.net. No permission is required from the copyright holders for the reproduction of this image. Points on the map were generated using Google Earth Pro and afterwords edited in Adobe^®^ Illustrator^®^ CS6 software.

Fifty-eight bacterial taxa were identified to best explain the observed microbial community differences among host ecomorphs (LEfSe, LDA > 3); seven were differentially more abundant on arboreal frogs, 39 were more abundant on terrestrial frogs, and 12 were more abundant on aquatic frogs (Supplementary Table 8). For example, the genus *Pigmentiphaga* (Alcaligenaceae) was significantly enriched on arboreal frogs, *Agrobacterium* (Rhizobiaceae) was significantly enriched on terrestrial frogs, and *Methylotenera* (Methylophilaceae) was significantly enriched on aquatic frogs (**Figure 3**). In particular, the relationship between *Pigmentiphaga* and arboreal frogs was seen across host genera (e.g., *Boophis* and *Heterixalus*) and locations (**Figure 4**).

**FIGURE 5:**
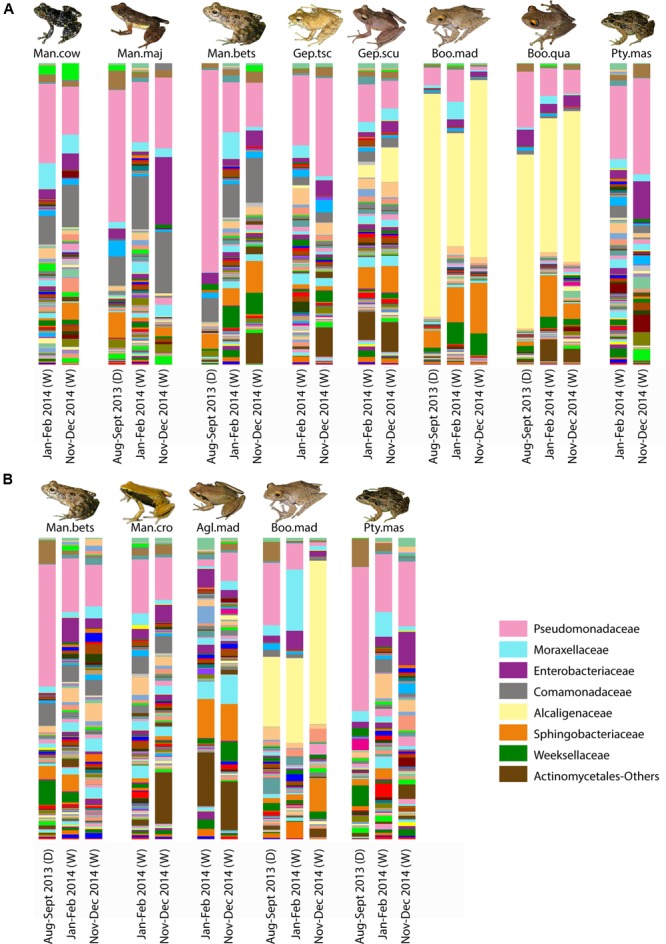
Variation of skin bacterial composition for selected frog species in Madagascar across time. Taxonomic composition of skin bacterial communities at the family level for selected host species from Ranomafana **(A)** and Andasibe **(B)** across the three sampling time points. Each bar represents the average community composition for a given species at the given sampling time. Sampling time is given below each bar; parenthetical “D” indicates dry season, and “W” represents wet season. Dominant taxa are identified in the bottom right. Frog species are abbreviated as follows: Man.cow, *Mantidactylus cowani* “small”; Man.maj, *Mantidactylus majori*; Man.bet, *Mantidactylus betsileanus*; Gep.tscsch, *Gephyromantis tschenki*; Gep.scu, *Gephyromantis sculpturatus*; Boo.mad, *Boophis madagascariensis*; Boo.qua, *Boophis quasiboehmei*; Pty.mad, *Ptychadena mascareniensis*; Man.cro, *Mantella crocea*; and Agl.mad, *Aglyptodactylus madagascariensis.* Inset frog photos were taken by M. Vences.

For selected frog hosts, variation through time was explored. While bacterial composition exhibited differences between sampling time points, key bacterial taxa [e.g., Alcaligenaceae in arboreal frogs (*Boophis*)] were consistently present through time (**Figure 5**).

Richness and diversity of frog skin microbial communities were also affected by multiple factors, with host ecomorphology exerting the strongest influence in most cases (**Table 2**). Host phylogeny and latitude also influenced species richness and diversity (**Table 2**). Pair-wise comparisons between host ecomorphs revealed that the main effect of host ecomorph was driven by arboreal frogs having significantly less diverse bacterial communities than both aquatic and terrestrial frogs, and aquatic frog having slightly less diverse communities than terrestrial frogs (**Tables 3, 4**).

**Table 2:** Generalized linear model results for the main factors influencing species richness and diversity indices of cutaneous bacterial communities on Malagasy amphibians.

		Richness or Diversity Index			
Factor	*DF*	Number of OTUs	Chao1	Effective number of species [exp(Shannon Index)]	Faith’s PD
Host ecomorph	2 980	**25.686** <0.01	**26.438** <0.01	**71.451** <0.01	21.339 <0.01
Latitude	21 980	21.781 <0.01	19.168 <0.01	6.293 0.012	**29.522** <0.01
Elevation	21 980	0.747 0.388	1.171 0.279	1.144 0.285	2.709 0.100
Host phylogeny (nMDS1)	21 980	8.899 0.003	11.907 0.001	2.262 0.133	7.932 0.005

*Wald chi-square values and *p*-values are presented. The strongest predictor is bolded.*

**Table 3:** Richness and diversity of cutaneous bacterial communities on Malagasy amphibians across the three ecomorph categories given as mean ± standard error.

	Amphibian ecomorph		
Richness/diversity metric	Arboreal	Aquatic	Terrestrial
Number of OTUs	167.5 ± 7.3	239.4 ± 14.6	277.1 ± 13.9
Chao1	203 ± 9.3	284.3 ± 18.6	335.6 ± 18.1
Effective number of species [exp(Shannon Index)]	188.3 ± 98.5	707.9 ± 25.8	784.9 ± 87.2
Faith’s phylogenetic diversity	31 ± 0.9	40.35 ± 1.8	43.5 ± 1.6

**Table 4:** Sidak *post hoc* test results for pair-wise comparisons between host ecomorph categories for richness and diversity values of Malagasy frog cutaneous bacterial communities.

	Richness or Diversity Index			
Comparison	Number of OTUs	Chao1	Effective number of species [exp(Shannon Index)]	Faith’s PD
Arboreal – Terrestrial	<0.01	<0.01	<0.01	<0.01
Arboreal – Aquatic	0.070	0.164	<0.01	0.017
Terrestrial – Aquatic	0.048	0.015	0.640	0.397

**FIGURE 6:**
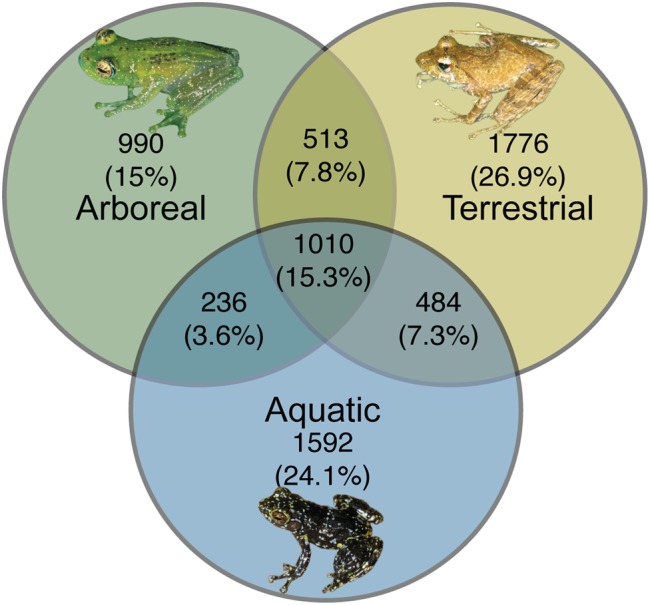
Unique and shared bacterial OTUs across ecomorphs of Malagasy frogs. Bacterial OTUs were considered present if it had a minimum of 5 reads in the rarified dataset. Inset frog photos were taken by M. Vences.

From a presence-absence perspective, there were 990 (15%) OTUs, 1592 (24.1%) OTUs, and 1776 (26.9%) OTUs (summed across all individuals of a given ecomorph) that were unique to arboreal, aquatic and terrestrial frogs, respectively. There were also OTUs shared between these groups; 15.3% of OTUs were shared across all ecomorphs, 7.8% of OTUs were shared between arboreal and terrestrial frogs, 7.3% were shared between terrestrial and aquatic frogs, and 3.6% were shared between aquatic and arboreal frogs (**Figure 6**).

## Discussion

The hyperdiverse amphibian communities of Madagascar offer a unique system for studying the factors that structure skin-associated microbial communities. This study is among the first to systematically explore amphibian skin microbiota on such a large geographical scale and to comparatively evaluate the roles of host phylogeny and host ecology.

Our results demonstrate that frogs skin microbiota differed from environmental substrates, suggesting that the skin is a unique niche in which only selected taxa can colonize and persist. In particular, Gammaproteobacteria were significantly enriched on frogs, while Alpha- and Betaproteobacteria were enriched in the environment. This distinct signature of the frog skin microbiota is in concordance with other studies that have found that skin communities are enriched for bacterial taxa that are in low relative abundance in the environment (Walke et al., 2014; Rebollar et al., 2016). The bacterial communities of Malagasy frog skin were predominantly composed of Proteobacteria, Bacteriodetes, Actinobacteria, Firmicutes, and Acidobacteria. This is similar to that found in other studied amphibians around the world (e.g., Kueneman et al., 2014; Belden et al., 2015; Sabino-Pinto et al., 2016), further suggesting that amphibian skin may act as a selective niche favoring particular taxa. Moreover, the high relative abundance of *Pseudomonas* on frog hosts mirrors the findings in other tropical regions, such as Panama (Belden et al., 2015; Rebollar et al., 2016). The fact that a unique community inhabits amphibian skin suggests host filtering is occurring, which could arise via multiple mechanisms including, host-produced compounds secreted into the skin mucosal environment (i.e., antimicrobial peptides, mucosal polysaccharides and proteins, or other metabolite-like compounds). Such compounds could differentially affect potential colonizers, by exhibiting both antimicrobial (i.e., inhibiting growth) or promicrobial (i.e., facilitating growth) activity (Conlon, 2011; Rollins-Smith and Woodhams, 2012; Franzenburg et al., 2013; Colombo et al., 2015).

Host ecomorphology was the strongest predictor of diversity and structure of cutaneous bacterial communities; therefore, ecological characteristics, including arboreality and association with water appear to be important drivers of variation in these communities. One possible explanation is that these microhabitat preferences expose frogs to different environmental microbial pools. Thus, while the skin microbiotas remain distinct from the environmental community, highlighting that a filtering process occurs, the structure and diversity of the surrounding environmental pool could affect colonization and succession dynamics of the skin community. Indeed, in a study on the salamander, *Plethodon cinereus*, the structure of the environmental microbial community largely affected the skin-associated microbial community structure (Loudon et al., 2014). Given the role of environmental transmission in maintenance of amphibian skin microbiota, the fact that environmental substrates differ in microbial composition (Fierer and Jackson, 2006; Hullar et al., 2006), could, in part, explain the host ecology effect. Alternatively, hosts with similar ecologies may be exposed to similar abiotic and biotic stressors. For example, arboreal frogs are more likely to be exposed to ultraviolet radiation. UV radiation has been shown to affect soil and aquatic environmental microbial communities (Jacobs and Sundin, 2001; Piccini et al., 2009; Hunting et al., 2013), and therefore, may also influence the microbial communities on frog skin. Moreover, from a biotic perspective, similar pathogen stressors are likely to be experienced by frogs with similar ecologies. For example, the ecological preferences of amphibian hosts have been related to their susceptibility to infection by the cutaneous pathogen, *Batrachochytrium dendrobatidis* (Stuart et al., 2004; Lips et al., 2006). Therefore, it is plausible that, over time, microbes that can offer protection against such pathogens would be selected for via changes in the chemical properties of the skin environment or production of specific defensive peptides. If such selective pressures are exerted more strongly on particular ecomorphs, this could drive the observed host ecology effect.

One of the most striking patterns associated with the ecomorphology effect, is the apparent association of bacteria from the genus *Pigmentiphaga* (Alcaligenaceae) with arboreal frog species. This genus was observed on arboreal frogs from multiple genera including, *Boophis, Heterixalus*, and *Spinomantis*, and was found on these frog genera across numerous locations and seasons. While little is known about the specific niches of Malagasy frogs, individuals from different species and genera spatially overlap and individuals of different species are often seen close to each other on the same leafs or in the same section of a small stream. Thus, occasional physical contact among non-conspecific frogs is likely, and horizontal transmission of skin bacteria is possible. Interestingly, another genus of Alcaligenaceae, *Achromobacter*, was strongly associated with the treefrogs, *Agalychnis callidryas* and *Dendropsophus ebraccatus* in Panama (Belden et al., 2015). This concordant finding in two distinct regions of the world, suggests that taxa from this family may have a particular facility for establishing on arboreal frogs and perhaps provide some beneficial function to their arboreal hosts. Not much is known about *Pigmentiphaga*; however, *Achromobacter* has been isolated from pine needles (Favilli and Messini, 1990), and in some plants, *Achromobacter* sp. confer tolerance to drought (Mayak et al., 2004).

While host phylogeny was found to be a significant factor in shaping microbial community structure (albeit less than host ecology) in model-based analyses (i.e., PERMANOVAs), we found no further evidence for strong phylosymbiosis in frog skin microbial communities; that is, the similarity of amphibian skin microbial communities did not parallel host phylogeny (Brooks et al., 2016). Phylosymbiosis has been documented in gut microbiota across multiple host clades including humans, mice and various insects (Brooks et al., 2016), but has not been explored with respect to skin microbial communities. Based on current knowledge, gut microbiota are more intimately linked to host processes, such as metabolism and immune system development (Ley et al., 2008; Chung et al., 2012; Sommer et al., 2016), For example, amphibians and other hosts with specialized diets have unique microorganisms enabling proper digestion and metabolism of particular compounds (Kohl et al., 2013, 2014; Vences et al., 2016); Thus, co-evolution patterns could be expected to occur more easily, driving the existence of phylosymbiosis patterns (Amato, 2013; Shapira, 2016). Apart from bacterial symbionts, a recent study found no association between amphibian phylogeny and eukaryotic parasites in the diverse South American amphibian fauna, and attributed this to a pattern whereby the majority of amphibian parasites are generalists (Campião et al., 2015). Thus, it is possible that specialist symbionts may be more likely to demonstrate phylosymbiosis with host taxa.

Elevation and latitude were also secondary factors that influenced the skin bacterial communities. These factors both represent site parameters suggesting that geographic location plays at least a small role in assembly of amphibian skin microbiota, which has also been found in previous studies on amphibian skin microbiota (Kueneman et al., 2014; Rebollar et al., 2016). Amphibian microbial communities are thought, at least in part, to be assembled and maintained via environmental transmission (Loudon et al., 2014), and environmental microbial communities are also known to vary across geographic space (Fierer and Jackson, 2006; Fierer, 2008). The sampled locations in Madagascar included rainforest sites at varying elevations, including, high-elevation montane sites, as well as semi-arid grasslands and canyon gallery forest sites; thus, differences in the environmental microbiota can be expected and may explain the observed effect of site-related parameters.

Overall, our findings illustrate that frog cutaneous microbiotas are distinct from the environment, and that while multiple factors influence the cutaneous microbial communities of amphibians, host ecomorphology is the main driver of cutaneous microbial diversity and structure in the biodiversity hotspot, Madagascar. For amphibians as well as other wildlife, microbes play an important role in mediating disease susceptibility. Gaining an understanding of the ecological forces structuring host-associated communities at different spatial scales provides a foundation for elucidating their role in host health and for understanding how these communities can be targeted with microbial therapies to promote positive health outcomes.

## Author Contributions

MCB, RNH, VM, and MV designed the project. MCB, RNH, FR, and AR collected field data. MCB and HA completed laboratory work. MCB and MV completed all data analysis. MCB wrote the paper. All authors contributed to revision of the manuscript and have approved the final manuscript.

## Conflict of Interest Statement

The authors declare that the research was conducted in the absence of any commercial or financial relationships that could be construed as a potential conflict of interest.

## Footnotes

1 https://github.com/biocore/deblur
