# Supplementary material for: Host Ecology Rather Than Host Phylogeny Drives Amphibian Skin Microbial Community Structure in the Biodiversity Hotspot of Madagascar
Source: Front Microbiol. 2017 Aug 17;8:1530. doi: 10.3389/fmicb.2017.01530 (PMC5563069; doi:10.3389/fmicb.2017.01530)
Supplement: Supplementary file 1 [file Table_1.pdf]

Host ecology rather than host phylogeny drives amphibian skin microbial community structure in the biodiversity hotspot of Madagascar

Molly C. Bletz[1*], Holly Archer[2], Reid N. Harris[3], Valerie McKenzie[2], Falitiana CE Rabemananjara[4], Andolalao Rakotoarison[1,4], Miguel Vences[1]

Supplementary Material

Supplementary Table 1. Sample sizes for each frog species and environmental substrate across sampled locations in Madagascar.

	**Location**									
**Host Species**	**Farankaraina**	**Fierenana**	**Andasibe**	**Antoetra**	**Ranomafana**	**Pic Ivohibe**	**Isalo**	**Andringitra**	**Ankaratra**	**Ambohitantely**
Aglyptodactylus madagascariensis	--	1	16	--	1	--	--	--	--	--
Anodonthyla boulengeri	8	--	2	--	12	2	--	--	--	--
Anodonthyla montana	--	--	--	--	--	--		6	--	--
Blommersia blommersae	1	1	10	--	6	--	--	--	--	3
Boophis albilabris	--	--	4	--	--	--	--	--	--	--
Boophis ankaratra	--	--	--	--	--	--	--	--	--	2
Boophis bottae	--	--	1	--	2	--	--	--	--	--
Boophis elenae	--	--	--	--	3	--	--	--	--	--
Boophis goudoti	--	--	2	3	--	--	--	3	10	4
Boophis guibei	--	1	3	--	6	--	--	--	--	--
Boophis idae	--	2	9	--	1	--	--	--	--	--
Boophis laurenti	--	--	--	--	--	--	--	8	--	--
Boophis luciae	--	--	--	--	10	--	--	--	--	--
Boophis luteus	--	--	3	--	4	--	--	--	--	--
Boophis madagascariensis	--	--	17	--	22	10	--	--	--	3
Boophis marojezensis	--	--	2	--	--	--	--	--	--	--
Boophis microtympanum	--	--	--	2	--		--	16	--	--
Boophis obscurus	--	--	--	--	--	6	1	--	--	--
Boophis picturatus	--	--	--	--	7	--	--	--	--	--
Boophis popi	--	--	--	--	--	2	--	7	--	--
Boophis pyrrhus	--	--	17	--	--	--	--	--	--	--
Boophis quasiboehmei	--	--	--	--	27	9	--	--	--	--
Boophis rappiodes	--	7	1	--	--	--	--	--	--	--
Boophis reticulatus	--	--	--	--	6	--	--	--	--	--
Boophis rhodoscelis	--		--	5	--	--	--	--	--	--
Boophis roseipalmatus	10	--	--	--	--	--	--	--	--	--

Boophis schuboeae	--	--	--	--	--	2	--	--	--	--
Boophis tasymena	--	--	6	--	2		--	--	--	--
Boophis tephraeomystax	2	--	--	--	--	--	--	--	--	--
Boophis viridis	--	--	10	--	--	--	--	--	--	--
Boophis williamsi	--	--	--	--	--	--	--	--	2	
Dyscophus antongilii	5	--	--	--	--	--	--	--	--	--
Dyscophus guineti	--	4	--		--	--		--	--	--
Gephyromantis asper	2	--	--	--	9		--	--	--	--
Gephyromantis blanci	--	--	--	--	--	3	--	--	--	--
Gephyromantis corvus	--	--	--	--	--	--	11		--	--
Gephyromantis decaryi	--	--	--	--	2	--	--	--	--	--
Gephyromantis enki	--	--	--	--	4	--	--	--	--	--
Gephyromantis leucomaculatus	7	--	--	--	--	--	--	--	--	--
Gephyromantis luteus	6	--	--	--	--	--	--	--	--	--
Gephyromantis sculpturatus	--	--	4	--	20	5	--	--	--	--
Gephyromantis silvanus	6	--	--	--	--	--	--	--	--	--
Gephyromantis tschenki	--	--	--	--	10	1	--	--	--	--
Gephyromantis webbi	7	--	--	--	--	--	--	--	--	--
Guibemantis depressiceps	--	--	--	--	5	--	--	--	--	--
Guibemantis flavobrunneus	--	--	2	--	--	--	--	--	--	--
Guibemantis liber	--	--	1	--	10	--	--	--	--	5
Guibemantis pulcher	--	--	--	--	3	--	--	--	--	--
Guibemantis timidus	3	--	1	--	--	--	--	--	--	--
Guibemantis tornieri	--	--	6	--	--	--	--	--	--	--
Heterixalus betsileo	--	11	5		15		--	--	5	--
Heterixalus madagascariensis	2	--	--	--	--	--	--	--	--	--
Heterixalus punctatus	4	--	5	--	--	--	--	--	--	--
Mantella aurantiaca	--	--	6	--	--	--	--	--	--	--
Mantella baroni	--		--	7	5	9	--	--	--	--
Mantella cowani	--		--	8	--	--	--	--	--	--
Mantella crocea	--	--	18	--	--	--	--	--	--	2
Mantella ebenaui	3	--	--	--	--	--	--	--	--	--
Mantella expectata	--	--	--	--	--	--	5	--	--	--
Mantella laevigata	5	--	--	--	--	--	--	--	--	--
Mantella milotympanum	--	10	--	--	--	--	--	--	--	--
Mantidactylus alutus	--	--	--	4	--	--	--	--	--	--
Mantidactylus argenteus	--	--	2	--	--	3	--	--	--	--
Mantidactylus betsileanus	15	--	20	--	12	2	--	--	--	--
Mantidactylus biporus	3	--	5	--	7	3	--	--		3
Mantidactylus bourgati	--	--	--	--	--	--	--	21	--	--
Mantidactylus charlotteae	3	--	--	--	5	--	--	--	--	--
Mantidactylus cowanii small	--	--	--	--	16	--	--	--	--	--
Mantidactylus curtus	--	--	--	2	--	--	--	--	--	--

Mantidactylus delormei	--	--	--	--	--	--	--	6	--	--
Mantidactylus femoralis	--	--	8	--	6	4	5	--	--	4
Mantidactylus grandidieri	--	--	1	--	5	1	--	--	--	4
Mantidactylus lugubris	--	--	4	5	--	10	--	--	--	--
Mantidactylus madecassus	--	--	--		--	--	--	4	--	--
Mantidactylus majori	--	--	--		12	--	--	--	--	--
Mantidactylus melanopleura	--	--	4	--	7	2	--	--	--	--
Mantidactylus mocquardi	--	--	--	--	5	--	--	--	--	--
Mantidactylus opiparis	--	--	--	--	1	--	--	--	--	3
Mantidactylus pauliani	--	--	--	--	--	--	--	--	7	--
Mantidactylus sp. 19 aff. Curtus	--	--	--	--	--	--	--	--	3	--
Mantidactylus sp. 57 aff. Grandidieri	7	--	--	--	--	--	--	--	--	--
Mantidactylus ulcerosus	--	--	--	--	--	--	2		--	--
Paradoxophyla palmata	--	--	3	--	--	--	--	--	--	--
Platypelis grandis	4	--	--	--	1	1	--	--	--	--
Platypelis pollicaris	--	--	3	--	8	--	--	--	--	2
Plethodontohyla mihanika	--	--	2	--	--	--	--	--	--	--
Plethodontohyla notosticta	10	--	--	--	--	--	--	--	--	--
Ptychadena mascareniensis	7	10	10		17	--	--	--	6	--
Scaphiophryne gottlebei	--	--	--	--	--	--	2	--	--	--
Scaphiophyrne marmorata	--	--	2	--	--	--	--	--	--	--
Spinomantis aglavei	--	--	5	--	4		--	--	--	--
Spinomantis bertini	--	--	--	--	--	10	--	--	--	--
Spinomantis elegans	--	--	--	--	--	4	--	--	--	--
Spinomantis fimbriatus	--	--	--	--	--	--	--	--		2
Spinomantis peraccae	--	--	--	--	--	3	--	--	--	5
Stumpffia tetradactyla	4	--	--	--	--	--	--	--	--	--
Water	--	--	--	--	2	2	2	1	--	--
Soil	--	--	2	--	3	5	2	4	--	--
Stream sediment	--	--	--	--	5	--	--	--	--	--
Leaf	--	--	2	--	1	1	--	--	--	--
