# Supplementary material for: Host Ecology Rather Than Host Phylogeny Drives Amphibian Skin Microbial Community Structure in the Biodiversity Hotspot of Madagascar
Source: Front Microbiol. 2017 Aug 17;8:1530. doi: 10.3389/fmicb.2017.01530 (PMC5563069; doi:10.3389/fmicb.2017.01530)
Supplement: Supplementary file 2 [file Table_2.pdf]

Host ecology rather than host phylogeny drives amphibian skin microbial community structure in the biodiversity hotspot of Madagascar

Molly C. Bletz[1*], Holly Archer[2], Reid N. Harris[3], Valerie McKenzie[2], Falitiana CE Rabemananjara[4], Andolalao Rakotoarison[1,4], Miguel Vences[1]

Supplementary Material

Supplementary Table 2. Latitude and longitude for each site within the 10 major locations at which sampling took place. Values presented in decimal degrees.

Region	Location	Site	Longitude	Latitude
Central Plateau	Antoetra	Ankaramasina	47.31591	-20.8066
		Faramivaza	47.33219	-20.82285
		Soamasaka	47.2952	-20.74795
	Andringitra	Base Camp Forest	46.94767	-22.14624
		Cuvette	X	X
		Plateau	46.94767	-22.14624
	Ankaratra	Village rice field	47.42566	-19.38136
		Ambohimirandrana	47.27428	-19.34079
		Tavolotara Camp	47.27705	-19.34634
	Ambohintantely	Lower Stream Site	47.2817	-18.19814
		Zone 3-Marsh	47.28122	-18.17243
North Central East	Andasibe	Mitsinjo Forest	48.41312	-18.93283
		Torotorofotsy Prolemur Camp	48.43222	-18.7709
		Voihmana	48.514732	-18.92085
	Fierenana	Beravotavo	48.43417	-18.60177
Northeast	Farankaraina	Farankaraina Station	49.83836	-15.43585
		Nosy Mangabe	49.76197	-15.49668
		Maroantsetra	49.4187	-15.43329
South Central East	Ranomafana	Ambatoladimy	47.42057	-21.25034
		Ambatolahy	47.42489	-21.24599
		Ranomafanakely	47.37186	-21.24866
		Talatakely	47.2216	-21.25553
		Vatoharanana	47.42963	-21.28882
		Vohiparara	47.39752	-21.23565
	Pic Ivohibe	Camp 3	46.95644	-22.49667
		Elegans Cave	46.95758	-22.5971
		Maravitsika	46.95644	-22.48239
West	Isalo	Zahavolo	45.3693	-22.61814
		Cayon de Rats	45.38118	-22.48119
		Malaso	45.35611	-22.59175
