# Supplementary material for: Host Ecology Rather Than Host Phylogeny Drives Amphibian Skin Microbial Community Structure in the Biodiversity Hotspot of Madagascar
Source: Front Microbiol. 2017 Aug 17;8:1530. doi: 10.3389/fmicb.2017.01530 (PMC5563069; doi:10.3389/fmicb.2017.01530)
Supplement: Supplementary file 3 [file Table_3.pdf]

Host ecology rather than host phylogeny drives amphibian skin microbial community structure in the biodiversity hotspot of Madagascar

Molly C. Bletz[1*], Holly Archer[2], Reid N. Harris[3], Valerie McKenzie[2], Falitiana CE Rabemananjara[4], Andolalao Rakotoarison[1,4], Miguel Vences[1]

Supplementary Material

Supplementary Table 3: Ecomorphological character state information determined from Glaw & Vences (2007).

Character description	Degree of arboreality	Degree of water-dependence	Reproductive mode	Egg deposition	Breeding water	Body size	Primary forest dependence
**Character type**	*ordered*	*ordered*	*unordered*	*unordered*	*unordered*	*ordered*	*ordered*
**0**	fully arboreal	fully or largely aquatic	amplexus	outside water	terrestrial	10-20 mm	typically outside forest
**1**	partly arboreal /scansorial	riparian/partly aquatic	no amplexus, eggs terrestrial	inside water	phytotelmes	20-30 mm	sometimes outside forest or forest edge
**2**	rarely scansorial	for reproduction in water	no amplexus, terrestrial nidicolous		stream	30-40 mm	typically in primary forest
**3**	fully terrestrial/ aquatic	almost never in water	no amplexus, eggs arboreal		pond	> 40 mm	
