# Supplementary material for: Host Ecology Rather Than Host Phylogeny Drives Amphibian Skin Microbial Community Structure in the Biodiversity Hotspot of Madagascar
Source: Front Microbiol. 2017 Aug 17;8:1530. doi: 10.3389/fmicb.2017.01530 (PMC5563069; doi:10.3389/fmicb.2017.01530)
Supplement: Supplementary file 4 [file Table_4.pdf]

Host ecology rather than host phylogeny drives amphibian skin microbial community structure in the biodiversity hotspot of Madagascar

Molly C. Bletz[1*], Holly Archer[2], Reid N. Harris[3], Valerie McKenzie[2], Falitiana CE Rabemananjara[4], Andolalao Rakotoarison[1,4], Miguel Vences[1]

Supplementary Material

Supplementary Table 4: Ecomorphological character states for each sampled frog species in Madagascar. Species within the Ranomafana subset are indicated with an asterisk.

Full dataset	Degree of arboreality	Degree of water-dependence	Reproductive mode	Egg deposition	Breeding water	Body size	Primary forest dependence
Aglyptodactylus madagascariensis *	2	2	0	1	3	3	2
Anodonthyla boulengeri *	0	2	0	1	1	0	1
Anodonthyla montana	3	2	0	0	1	2	0
Blommersia blommersae *	1	3	3	0	3	0	1
Boophis albilabris	0	2	0	1	2	3	2
Boophis bottae *	0	2	0	1	2	1	2
Boophis elenae *	0	2	0	1	2	2	2
Boophis goudoti	1	1	0	1	3	3	1
Boophis guibei *	0	2	0	1	3	2	2
Boophis idae *	0	2	0	1	3	2	2
Boophis laurenti	1	1	0	1	2	2	0
Boophis luciae *	0	2	0	1	2	2	2
Boophis luteus *	0	2	0	1	2	2	1
Boophis madagascariensis *	0	1	0	1	3	3	1
Boophis microtympanum	1	1	0	1	2	2	0
Boophis obscurus	1	1	0	1	3	3	1
Boophis picturatus *	0	2	0	1	2	2	2
Boophis popi	0	2	0	1	2	1	2
Boophis pyrrhus	0	2	0	1	2	1	1
Boophis quasiboehmei *	0	2	0	1	2	1	2
Boophis rappiodes	0	2	0	1	2	1	2
Boophis reticulatus *	0	2	0	1	2	2	2
Boophis rhodoscelis	0	2	0	1	2	1	1
Boophis roseipalmatus	0	2	0	1	3	3	2
Boophis schuboeae	0	2	0	1	2	2	2
Boophis tasymena *	0	2	0	1	2	1	2
Boophis viridis	0	2	0	1	2	2	1
Dyscophus antongilii	3	2	0	1	3	3	0

Dyscophus guineti	3	2	0	1	3	3	1
Gephyromantis asper *	1	3	1	0	2	1	2
Gephyromantis blanci	1	3	2	0	0	0	2
Gephyromantis corvus	1	3	1	0	2	2	2
Gephyromantis enki *	1	3	2	0	0	0	2
Gephyromantis leucomaculatus	1	3	1	0	2	2	2
Gephyromantis luteus	1	3	1	0	2	2	2
Gephyromantis sculpturatus *	1	3	1	0	2	2	2
Gephyromantis silvanus	2	3	1	0	2	2	2
Gephyromantis tschenki *	1	3	1	0	2	2	2
Gephyromantis webbi	2	3	1	0	2	1	2
Guibemantis depressiceps *	0	3	3	0	3	2	1
Guibemantis flavobrunneus	1	2	3	0	1	1	2
Guibemantis liber *	1	3	3	0	3	1	1
Guibemantis pulcher *	1	2	3	0	1	1	2
Guibemantis tornieri	0	3	3	0	3	2	1
Heterixalus betsileo *	0	2	0	1	3	1	0
Heterixalus punctatus	0	2	0	1	3	1	0
Mantella aurantiaca	3	3	1	0	3	1	2
Mantella baroni *	2	3	1	0	2	1	2
Mantella cowani	3	3	1	0	2	1	1
Mantella crocea	3	3	1	0	3	1	2
Mantella ebenaui	3	3	1	0	2	1	1
Mantella expectata	3	3	1	0	2	1	1
Mantella laevigata	1	2	3	0	1	1	2
Mantella milotympanum	3	3	1	0	3	1	2
Mantidactylus alutus	3	0	1	0	2	1	1
Mantidactylus argenteus	1	1	3	0	2	1	2
Mantidactylus betsileanus	2	1	1	0	2	1	1
Mantidactylus biporus *	3	0	1	0	2	1	2
Mantidactylus bourgati	3	0	1	0	2	2	1
Mantidactylus charlotteae *	2	1	1	0	2	2	2
Mantidactylus cowanii small *	2	0	1	0	2	2	2
Mantidactylus delormei	2	1	1	0	2	1	1
Mantidactylus femoralis *	2	1	3	0	2	2	2
Mantidactylus grandidieri *	3	0	1	0	2	3	2
Mantidactylus lugubris	2	0	1	0	2	2	2
Mantidactylus madecassus	3	0	1	0	2	2	0
Mantidactylus majori *	2	0	3	0	2	2	2
Mantidactylus melanopleura *	2	1	1	0	2	2	2
Mantidactylus mocquardi *	2	1	3	0	2	3	2
Mantidactylus opiparis *	2	1	1	0	2	2	2
Mantidactylus pauliani	3	0	1	0	2	2	0
Mantidactylus sp. 19 aff curtus	3	0	1	0	2	2	1

Mantidactylus sp. 57 aff grandidieri	3	0	1	0	2	3	2
Mantidactylus ulcerosus	3	1	1	0	2	2	1
Paradoxophyla palmata	3	2	0	1	3	1	2
Platypelis grandis *	0	2	0	1	1	3	2
Platypelis pollicaris *	0	2	0	1	1	1	2
Plethodontohyla mihanika *	1	2	0	1	1	2	2
Plethodontohyla notosticta	1	2	0	1	1	2	2
Ptychadena mascareniensis *	3	1	0	1	3	2	0
Scaphiophryne gottlebei	2	2	0	1	3	2	1
Scaphiophyrne marmorata	2	2	0	1	3	2	2
Spinomantis aglavei *	0	3	3	0	2	2	2
Spinomantis bertini	2	3	3	0	2	1	2
Spinomantis elegans	2	3	3	0	2	3	2
Spinomantis fimbriatus	0	3	3	0	2	2	2
Spinomantis peraccae	0	3	3	0	2	2	2
Stumpffia tetradactyla	3	3	2	0	0	0	1
