# Supplementary material for: Host Ecology Rather Than Host Phylogeny Drives Amphibian Skin Microbial Community Structure in the Biodiversity Hotspot of Madagascar
Source: Front Microbiol. 2017 Aug 17;8:1530. doi: 10.3389/fmicb.2017.01530 (PMC5563069; doi:10.3389/fmicb.2017.01530)
Supplement: Supplementary file 5 [file Table_5.pdf]

Host ecology rather than host phylogeny drives amphibian skin microbial community structure in the biodiversity hotspot of Madagascar

Molly C. Bletz[1*], Holly Archer[2], Reid N. Harris[3], Valerie McKenzie[2], Falitiana CE Rabemananjara[4], Andolalao Rakotoarison[1,4], Miguel Vences[1]

Supplementary Material

Supplementary Table 5. LEfSe-identified bacterial taxa exhibiting significant differential relative abundance between frog skin and environmental substrates in Madagascar.

Environment			
**Taxa**	**LDA**	**Taxa**	**LDA**
Acidobacteria	4.98	Planctomycetes	4.18
Acidobacteria_6	3.94	Planctomycetes, Planctomycetia	4.06
Acidobacteria, Acidobacteria_6, iii1_15	3.94	Planctomycetes, Planctomycetia, Gemmatales	4.06
Acidobacteria, Acidobacteriia	4.60	Planctomycetes, Planctomycetia, Gemmatales, Gemmataceae	3.91
Acidobacteria, Acidobacteriia, Acidobacteriales	4.60	Planctomycetes, Planctomycetia, Gemmatales, Gemmataceae, *Gemmata*	3.15
Acidobacteria, Acidobacteriia, Acidobacteriales, Koribacteraceae	4.48	Planctomycetes, Planctomycetia, Gemmatales, Isosphaeraceae	3.54
Acidobacteria, DA052	4.41	Proteobacteria, Alphaproteobacteria	4.94
Acidobacteria, DA052, Ellin6513	4.41	Proteobacteria, Alphaproteobacteria, Rhizobiales	4.64
Acidobacteria, Solibacteres, Solibacterales, Solibacteraceae, *Candidatus Solibacter*	3.84	Proteobacteria, Alphaproteobacteria, Rhizobiales, Bradyrhizobiaceae	3.79
Actinobacteria, Actinobacteria, Actinomycetales, Mycobacteriaceae	3.41	Proteobacteria, Alphaproteobacteria, Rhizobiales, Bradyrhizobiaceae, *Bradyrhizobium*	3.79
Actinobacteria, Actinobacteria, Actinomycetales, Mycobacteriaceae, *Mycobacterium*	3.41	Proteobacteria, Alphaproteobacteria, Rhizobiales, Hyphomicrobiaceae	4.19
Actinobacteria, Thermoleophilia	3.17	Proteobacteria, Alphaproteobacteria, Rhizobiales, Hyphomicrobiaceae, *Rhodoplanes*	4.19
Actinobacteria, Thermoleophilia, Solirubrobacterales	3.17	Proteobacteria, Alphaproteobacteria, Rhodospirillales, Rhodospirillaceae	4.09
Bacteroidetes, Saprospirae	4.23	Proteobacteria, Deltaproteobacteria	4.43
Bacteroidetes, Saprospirae, Saprospirales	4.23	Proteobacteria, Deltaproteobacteria, Myxococcales	4.17
Bacteroidetes, Saprospirae, Saprospirales, Chitinophagaceae	4.23	Proteobacteria, Deltaproteobacteria, Syntrophobacterales	3.99
Bacteroidetes, Cytophagia	3.79	Proteobacteria, Deltaproteobacteria, Syntrophobacterales, Syntrophobacteraceae	3.99

Bacteroidetes, Cytophagia, Cytophagales	3.79	Proteobacteria, Gammaproteobacteria, Xanthomonadales, Sinobacteraceae	4.29
Bacteroidetes, Cytophagia, Cytophagales, Cytophagaceae	3.79	Verrucomicrobia, Spartobacteria	4.11
Bacteroidetes, Flavobacteriia, Flavobacteriales, Flavobacteriaceae	3.46	Verrucomicrobia, Spartobacteria, Chthoniobacterales	4.11
Bacteroidetes, Flavobacteriia, Flavobacteriales, Flavobacteriaceae, *Flavobacterium*	3.46	Verrucomicrobia, Spartobacteria, Chthoniobacterales, Chthoniobacteraceae	4.11
Chloroflexi	4.21	Verrucomicrobia, Spartobacteria, Chthoniobacterales, Chthoniobacteraceae, DA101	3.96
Chloroflexi, Ktedonobacteria	4.21		
Chloroflexi, Ktedonobacteria, Thermogemmatisporales	4.21		
Chloroflexi, Ktedonobacteria, Thermogemmatisporales, Thermogemmatisporaceae	4.21		
**Frog**			
**Taxa**	**LDA**	**Taxa**	**LDA**
Firmicutes, Bacilli, Bacillales, Staphylococcaceae	3.92	Proteobacteria, Gammaproteobacteria, Enterobacteriales, Enterobacteriaceae	4.58
Firmicutes, Bacilli, Bacillales, Staphylococcaceae, *Staphylococcus*	3.92	Proteobacteria, Gammaproteobacteria, Enterobacteriales, Enterobacteriaceae, *Klebsiella*	4.34
Firmicutes, Bacilli, Lactobacillales	3.46	Proteobacteria, Gammaproteobacteria, Pseudomonadales	5.25
Firmicutes, Bacilli, Lactobacillales, Streptococcaceae	3.35	Proteobacteria, Gammaproteobacteria, Pseudomonadales, Pseudomonadaceae	5.19
Proteobacteria, Gammaproteobacteria	5.30	Proteobacteria, Gammaproteobacteria, Pseudomonadales, Pseudomonadaceae, *Pseudomonas*	5.16
Proteobacteria, Gammaproteobacteria, Enterobacteriales	4.58		
