# Supplementary material for: Host Ecology Rather Than Host Phylogeny Drives Amphibian Skin Microbial Community Structure in the Biodiversity Hotspot of Madagascar
Source: Front Microbiol. 2017 Aug 17;8:1530. doi: 10.3389/fmicb.2017.01530 (PMC5563069; doi:10.3389/fmicb.2017.01530)
Supplement: Supplementary file 6 [file Table_6.pdf]

Host ecology rather than host phylogeny drives amphibian skin microbial community structure in the biodiversity hotspot of Madagascar

Molly C. Bletz[1*], Holly Archer[2], Reid N. Harris[3], Valerie McKenzie[2], Falitiana CE Rabemananjara[4], Andolalao Rakotoarison[1,4], Miguel Vences[1]

Supplementary Material

Supplementary Table 6. Results of Multivariate Regression on distance Matrices for host ecomorphologial matrices of Malagasy frogs derived from two, three, four, and seven characters. Each model included the respective ecomorphology matrix and the host phylogenetic matrix derived from patristic distances between species of Malagasy frogs. P-values for both matrices are provided from both the full dataset and Ranomafana subset only.

		**Host ecomorphological matrix type**			
	**# of characters**	**2**	**3**	**4**	**7**
Dataset	Full data	Eco: 0.562<br>Phylo:0.349	Eco: 0.509<br>Phylo:0.377	Eco: 0.484<br>Phylo:0.386	Eco: 0.967<br>Phylo:0.338
	Ranomafana	Eco: 0.114<br>Phylo:0.519	Eco: 0.029<br>Phylo:0.579	Eco: 0.021<br>Phylo:0.700	Eco: 0.927<br>Phylo:0.660
