# Supplementary material for: Host Ecology Rather Than Host Phylogeny Drives Amphibian Skin Microbial Community Structure in the Biodiversity Hotspot of Madagascar
Source: Front Microbiol. 2017 Aug 17;8:1530. doi: 10.3389/fmicb.2017.01530 (PMC5563069; doi:10.3389/fmicb.2017.01530)
Supplement: Supplementary file 7 [file Table_7.pdf]

Host ecology rather than host phylogeny drives amphibian skin microbial community structure in the biodiversity hotspot of Madagascar

Molly C. Bletz[1*], Holly Archer[2], Reid N. Harris[3], Valerie McKenzie[2], Falitiana CE Rabemananjara[4], Andolalao Rakotoarison[1,4], Miguel Vences[1]

Supplementary Material

Supplementary Table 7. Average pairwise weighted unifrac distances within and between the microbial communities of amphibian ecomorphs in Madagascar. Between-ecomorph values are highlighted in grey.

	**Terrestrial**	**Aquatic**	**Arboreal**
**Terrestrial**	0.70 ± 0.2		
**Aquatic**	0.73 ± 0.2	0.75 ± 0.2	
**Arboreal**	0.76 ± 0.23	0.77 ± 0.23	0.72 ± 0.3
