# Supplementary material for: Host Ecology Rather Than Host Phylogeny Drives Amphibian Skin Microbial Community Structure in the Biodiversity Hotspot of Madagascar
Source: Front Microbiol. 2017 Aug 17;8:1530. doi: 10.3389/fmicb.2017.01530 (PMC5563069; doi:10.3389/fmicb.2017.01530)
Supplement: Supplementary file 8 [file Table_8.pdf]

Host ecology rather than host phylogeny drives amphibian skin microbial community structure in the biodiversity hotspot of Madagascar

Molly C. Bletz[1*], Holly Archer[2], Reid N. Harris[3], Valerie McKenzie[2], Falitiana CE Rabemananjara[4], Andolalao Rakotoarison[1,4], Miguel Vences[1]

Supplementary Material

Supplementary Table 8. LEfSe-identified bacteria taxa exhibiting differential relative abundance between frog ecomorphs from Madagascar.

Taxa	LDA
**Aquatic**	
**Taxa**	**LDA**
Bacteroidetes, Flavobacteriia, Flavobacteriales, Flavobacteriaceae	3.74
Bacteroidetes, Flavobacteriia, Flavobacteriales, Flavobacteriaceae, *Flavobacterium*	3.74
Proteobacteria, Betaproteobacteria, Burkholderiales, Comamonadaceae	4.49
Proteobacteria, Betaproteobacteria, Methylophilales	3.45
Proteobacteria, Betaproteobacteria, Methylophilales, Methylophilaceae	3.40
Proteobacteria, Betaproteobacteria, Methylophilales, Methylophilaceae, *Methylotenera*	3.42
Proteobacteria, Deltaproteobacteria, Myxococcales, Myxococcaceae	3.28
Proteobacteria, Deltaproteobacteria, Myxococcales, Myxococcaceae, Anaeromyxobacter	3.29
Verrucomicrobia	3.93
Verrucomicrobia, Verrucomicrobiae	3.87
Verrucomicrobia, Verrucomicrobiae, Verrucomicrobiales	3.86
Verrucomicrobia, Verrucomicrobiae, Verrucomicrobiales, Verrucomicrobiaceae	3.86
**Arboreal**	
**Taxa**	**LDA**
Actinobacteria, Actinobacteria, Actinomycetales, Microbacteriaceae	3.29
Bacteroidetes, Sphingobacteriia, Sphingobacteriales, Sphingobacteriaceae	4.09
Proteobacteria	4.53
Proteobacteria, Betaproteobacteria	4.98
Proteobacteria, Betaproteobacteria, Burkholderiales	4.98
Proteobacteria, Betaproteobacteria, Burkholderiales, Alcaligenaceae	5.04
Proteobacteria, Betaproteobacteria, Burkholderiales, Alcaligenaceae, *Pigmentiphaga*	5.04
**Terrestrial**	
**Taxa**	**LDA**
Acidobacteria	4.05
Acidobacteria, Acidobacteriia	3.64
Acidobacteria, Acidobacteriia, Acidobacteriales	3.64
Acidobacteria, Acidobacteriia, Acidobacteriales, Acidobacteriaceae	3.37
Acidobacteria, Acidobacteriia, Acidobacteriales, Koribacteraceae, *Candidatus Koribacter*	3.06
Acidobacteria, DA052	3.53
Acidobacteria, DA052, Ellin6513	3.49

Acidobacteria, Solibacteres	3.52
Acidobacteria, Solibacteres, Solibacterales	3.52
Acidobacteria, Solibacteres, Solibacterales, Solibacteraceae	3.32
Acidobacteria, Solibacteres, Solibacterales, Solibacteraceae, *Candidatus Solibacter*	3.06
Actinobacteria, Actinobacteria, Actinomycetales, Frankiaceae	3.23
Actinobacteria, Actinobacteria, Actinomycetales, Pseudonocardiaceae	3.36
Actinobacteria, Actinobacteria, Actinomycetales, Pseudonocardiaceae, *Pseudonocardia*	3.35
Planctomycetes	3.91
Planctomycetes, Phycisphaerae	3.42
Planctomycetes, Phycisphaerae, WD2101	3.35
Planctomycetes, Planctomycetia	3.71
Planctomycetes, Planctomycetia, Gemmatales	3.71
Planctomycetes, Planctomycetia, Gemmatales, Gemmataceae, *Gemmata*	2.87
Proteobacteria, Alphaproteobacteria	4.46
Proteobacteria, Alphaproteobacteria, Ellin329	3.51
Proteobacteria, Alphaproteobacteria, Rhizobiales	4.17
Proteobacteria, Alphaproteobacteria, Rhizobiales, Bradyrhizobiaceae	3.48
Proteobacteria, Alphaproteobacteria, Rhizobiales, Bradyrhizobiaceae, *Bradyrhizobium*	3.48
Proteobacteria, Alphaproteobacteria, Rhizobiales, Rhizobiaceae	3.78
Proteobacteria, Alphaproteobacteria, Rhizobiales, Rhizobiaceae, *Agrobacterium*	3.68
Proteobacteria, Alphaproteobacteria, Rhizobiales, Rhizobiaceae, *Rhizobium*	3.59
Proteobacteria, Alphaproteobacteria, Rhodospirillales	3.80
Proteobacteria, Alphaproteobacteria, Rhodospirillales, Rhodospirillaceae	3.70
Proteobacteria, Alphaproteobacteria, Sphingomonadales	3.74
Proteobacteria, Alphaproteobacteria, Sphingomonadales, Sphingomonadaceae	3.74
Proteobacteria, Alphaproteobacteria, Sphingomonadales, Sphingomonadaceae, *Novosphingobium*	3.30
Proteobacteria, Alphaproteobacteria, Sphingomonadales, Sphingomonadaceae, *Sphingomonas*	3.33
Proteobacteria, Betaproteobacteria, Burkholderiales, Comamonadaceae, *Comamonas*	3.26
Verrucomicrobia, Spartobacteria	3.60
Verrucomicrobia, Spartobacteria, Chthoniobacterales	3.49
Verrucomicrobia, Spartobacteria, Chthoniobacterales, Chthoniobacteraceae	3.60
Verrucomicrobia, Spartobacteria, Chthoniobacterales, Chthoniobacteraceae, *DA101*	3.08
